# Health Synergies across International Sustainability and Development Agendas: Pathways to Strengthen National Action

**DOI:** 10.3390/ijerph18041664

**Published:** 2021-02-09

**Authors:** Kathryn J. Bowen, Nabreesa Murphy, Sarah Dickin, Adis Dzebo, Charles Ebikeme

**Affiliations:** 1 Institute for Advanced Sustainability Studies, D-14467 Potsdam, Germany; 2 Fenner School of Environment and Society, Australian National University, Canberra 0200, Australia; 3 School of Population and Global Health, University of Melbourne, Melbourne 3010, Australia; nmurphy@ippf.org; 4 International Planned Parenthood Federation (IPPF), Australia and New Zealand Office, Melbourne 3000, Australia; 5 Stockholm Environment Institute, Linnégatan 87D, 115 23 Stockholm, Sweden; sarah.dickin@sei.org (S.D.); a.dzebo@uu.nl (A.D.); 6 Copernicus Institute of Sustainable Development, Utrecht University, Princetonlaan 8a, 3584 CB Utrecht, The Netherlands; 7 Department of Health Policy, London School of Economics and Political Science, London WC2A 2AE, UK; C.Ebikeme@lse.ac.uk

**Keywords:** global health, implementation, governance, climate change, urbanisation, disaster risk reduction, sustainable development, international agendas

## Abstract

Since 2015 there has been a surge of international agendas to address a range of global challenges: climate change (Paris Agreement), sustainable development (Agenda 2030), disaster risk reduction (Sendai Framework) and sustainable urban transformation (New Urban Agenda). Health is relevant to all of these agendas. Policymakers must now translate these global agendas into national level policies to implement the agreed goals in a coherent manner. However, approaches to synergise health activities within and across these agendas are needed, in order to achieve better coherence and maximise national level implementation. This research evaluated the framing of human health within these agendas. A content analysis of the agendas was conducted. Findings indicate (i) the importance of increased awareness of health systems strengthening as a helpful framework to guide the integration of health issues across the agendas, (ii) only two health themes had synergies across the agendas, (iii) the lack of a governance mechanism to support the integration of these four agendas to enable national (and sub-national) governments to more feasibly implement their ambitions, and (iv) the vital component of health leadership. Finally, planetary health is a relevant and timely concept that can support the urgent shift to a healthy planet and people.

## 1. Introduction

Disruptions to ecological and biophysical systems (air, land, water) via human exploitation of the environment have profound implications for planetary health—Defined as “the health of human civilisation and the state of the natural systems on which it depends” [1]. In this age of the Anthropocene, we are now able to directly observe the impact of human-led environmental upheaval on a global scale. Threats to our vital operating systems, or ‘planetary boundaries’ [2,3] expose populations to a greater risk of negative health outcomes, most of which are inequitably distributed. Efforts have increased in the last five years to respond to the variety of challenges confronting human society in order to secure a just and healthy future; the proliferation of international frameworks, agreements and agendas has demonstrated this increased effort, building on from the Millennium Development Goals (MDGs) and the Millennium Ecosystem Assessment [4]. The global attention on international agendas has reinvigorated focus and action and presents opportunities to respond to a range of enormous challenges—Including climate change (Paris Agreement) [5], sustainable development (Sustainable Development Goals Agenda 2030) [6], disaster risk reduction (Sendai Framework) [7] and rapid urbanisation (New Urban Agenda) [8].

In no other time in recent history has such a plethora of international agendas with almost universal support come together to create a momentum to develop and implement solutions to these urgent challenges. However, these global platforms have more to do than just providing clear and agreed upon policy direction—They must now be interpreted into regional, national, and sub-national programs in each signatory country. This is particularly important as these global agreements have been discussed and agreed upon with limited interaction between the separate regimes and United Nations agencies despite being ratified by the majority of UN member states. This points to a fragmentation of the landscape and an overall siloed approach by governments. Yet, the importance of understanding the implementation potential of these agendas is now of utmost concern. This is acutely relevant for the health sector, as the link between health and sustainable development has been widely documented. Health is not only a result of development, but health is a pre-requisite and driver of development. This has clear relevance to these agendas, either directly, or indirectly via health determining sectors—Such as agriculture, development, water, environment, urban planning and design.

Recently, gains have been made to promote effective ‘Health in All Policies’ approaches to public policies across sectors that systematically takes into account the health implications of decisions, seeks synergies, and avoids harmful health impacts in order to improve population health and health equity [9,10]. There are clear implications for global health outcomes depending on how these agendas are operationalised and implemented. This also points to the need for a coherent approach across the health sector and health advocacy groups. However, with the suite of post-2015 agendas, little has been done to provide a comprehensive global overview of coherence around health goals.

Considerable efforts have recently been made to better understand the policy coherence between two of these four agendas—The 2030 Agenda and the Paris Agreement (e.g., [11,12]). For example, the NDC-SDG Connections tool analysed countries Nationally Determined Contributions (NDCs), i.e., national implementation plans of the Paris Agreement, and mapped intended activities to the 17 SDGs [13]. With regard to the health sector specifically, Dickin and Dzebo [14] wrote that although the health sector seems on the surface to be well integrated into countries’ NDCs, concrete steps are missing. Beyond this, research has not extended to examine the links across all four agendas, and not yet used health as a framing. This paper aims to fill this gap.

Policy coherence for sustainable development is an established policy and research area [15,16], with multiple guidelines available on how to support the development of mutually reinforcing policies across different sectors to achieve goals and objectives (e.g., [17,18,19,20,21,22,23]). However, there has not yet been a sectoral assessment on the degree to which these international agreements do indeed demonstrate coherence in the context of health. Many tools are suggested to develop policy coherence, including quantitative modelling tools, Theory of Change and Multi-criteria Analysis (MCA), and analysis of systemic linkages and interdependencies among policy approaches (UN DESA). This paper evaluates the degree to which health is articulated across these four agendas, to identify synergies between them, as well as areas for improved cohesion to support their national implementation. A brief overview of each of the agendas is provided below.

### 1.1. Paris Agreement

The United Nations Framework Convention on Climate Change (UNFCCC) Paris Agreement is the first global comprehensive climate agreement. It became effective in late 2016 and has been ratified by 196 countries. Through the Paris Agreement, countries have agreed to (i) holding the increase in the global average temperature to well below 2 °C above pre-industrial levels and to pursue efforts to limit the temperature increase to 1.5 °C above pre-industrial levels, recognizing that this would significantly reduce the risks and impacts of climate change; (ii) increasing the ability to adapt to the adverse impacts of climate change and foster climate resilience and low greenhouse gas emissions development, in a manner that does not threaten food production; and (iii) making finance flows consistent with a pathway towards low greenhouse gas emissions and climate-resilient development. The Agreement commits to mobilising US$100 billion/year, from 2020 and onwards, in climate finance for vulnerable countries to implement mitigation and adaptation activities.

The relevance of the Paris Agreement to individual countries is through the NDCs, which are contributions that each individual country will make to achieve the global target. However, the contributions are not binding, and there are no mechanisms to force countries to set targets by certain timelines. Instead, countries are expected to ratchet-up their commitments in updated NDCs every 5 years [24]. Given that there are no consequences to meeting or failing to meet contributions determined by national governments, it is unclear how progress will occur, until 2023, when there will be a first a five-yearly collective analysis and evaluation of implementation efforts [25].

The Agreement specifically refers to health in two sections, first in the Preamble—“recognising also that when developing policies and taking action to address climate change, Parties should promote, protect, respect and take into account their respective obligations on all human rights, *the right to health*…” and also in the section Enhanced Action Prior to 2020—Recognizes the social, economic and environmental value of voluntary mitigation actions and their co-benefits for adaptation, *health and sustainable development*…”. Although the agreement makes reference to health, it falls short of unpacking its implications to climate change or vice versa.

### 1.2. 2030 Agenda

The 2030 Agenda for Sustainable Development was agreed by the United Nations General Assembly in 2015. It encompasses 17 Sustainable Development Goals (SDGs), 169 targets and a declaration text articulating the principles of integration, universality, transformation and a global partnership. The agenda came into being through a unique global process of an open working group, which jointly developed the 17 SDGs that were subsequently agreed on by all UN member states [26]. The SDGs include the social, environmental and economic dimensions of development. They aim to provide a social foundation for humanity while ensuring that human development takes place within earth’s biophysical boundaries [2]. The SDGs were developed to succeed the MDGs, which ended in 2015. The main differences between these two sets of development goals are that (i) the SDGs took a more global approach, with global surveys, reports and consultations producing much of the priority-setting process; (ii) they had different aims, encompassing the three dimensions of sustainable development: the economic, social and environmental, with the SDGs aiming to transform health, development and environmental standards not just for low and middle-income countries but for every country; and (iii) a large increase in the number of goals and targets.

At the national level, implementation of the 2030 Agenda varies from country to country, and is based on national needs and ambitions. At the international level, the High-Level Political Forum (HLPF) meets annually under the auspices of the UN Economic and Social Council (ECOSOC) to discuss Voluntary National Reviews (VNRs) as part of the official follow-up and review mechanism of the 2030 Agenda [26]. However, individual countries are left to set-up an institutional architecture for implementing the SDGs at national and subnational levels through National Sustainable Development Strategies.

The 2030 Agenda has one explicit health goal (SDG 3) “*Good Health and Well-Being*”, which includes nine targets. In addition, several other SDGs address underlying determinants of health. These include, inter alia, hunger (SDG 1), food (SDG 2), gender equality (SDG 5), water and sanitation (SDG 6), and reduced inequalities (SDG 10).

### 1.3. Sendai Framework for Disaster Risk Reduction 2015–2030

The Sendai Framework was adopted by UN member states in 2015, following the expiration of the Hyogo Framework for Action (2005–2015). The Sendai Framework has four specific priorities for action: (i) understanding disaster risk; (ii) strengthening disaster risk governance to manage disaster risk; (iii) investing in disaster risk reduction for resilience; and (iv) enhancing disaster preparedness for effective response, and to “Build Back Better” in recovery, rehabilitation and reconstruction.

Compared to its predecessor, the Sendai Framework states a “more explicit focus on people and their health and livelihoods”, and health is referenced in the framework’s aim of “the substantial reduction of disaster risks and losses in lives, livelihoods and health and in the physical, social, cultural and environmental assets of persons, businesses, communities and countries” over the next 15 years. The Framework also states that in order to achieve this outcome, the goal must be “Prevent new and reduce existing disaster risk through the implementation of integrated and inclusive economic, structural, legal, social, health, cultural, educational, environmental, technological, political and institutional measures that prevent and reduce hazard exposure and vulnerability to disaster, increase preparedness for response and recovery, and thus strengthen resilience”.

The assessment of global progress in achieving the outcome and goal of the Framework is supported by seven global targets, which are complemented by national targets and nationally determined indicators.

### 1.4. New Urban Agenda

The New Urban Agenda was endorsed by the UN General Assembly in 2016 and adopted by 167 countries. It provides global principles, policies and standards to guide the achievement of sustainable urban development over the next 20 years. The New Urban Agenda embodies three guiding principles: (i) leave no one behind, ensure urban equity and eradicate poverty, (ii) achieve sustainable and inclusive urban prosperity and opportunities for all, and (iii) foster ecological and resilient cities and human settlements.

The New Urban Agenda acknowledges the milestone achievements of 2015, particularly the SDGs, Paris Agreement and the Sendai Framework for Disaster Risk Reduction. SDGs are specifically mentioned as being complementary to the New Urban Agenda, stating that *“the implementation of the New Urban Agenda contributes to the implementation and localization of the 2030 Agenda and Sustainable Development…including Goal 11 of making cities and human settlements inclusive, resilient, safe and sustainable”*.

Unlike the SDGs, the New Urban Agenda does not provide indicators against which progress can be measured. Instead it recommends that the follow-up and review of the New Urban Agenda be closely aligned with the follow-up and review of the 2030 Agenda for Sustainable Development. This largely leaves the implementation and decisions on indicators to the discretion of local governments. Health is mentioned several times under a broad description of *“human health and well-being”*, in relation to the promotion of *“safe, inclusive, accessible, green and quality public spaces”*.

## 2. Materials and Methods

Qualitative content analysis was used to analyse all four policy agreement documents using MaxQDA software. The aim of the analysis was to identify all mentions of health in each agenda text, to compare between texts and identify commonalities, and to identify any reference to each other between the agendas. The process involved an iterative approach, where the coding framework was developed and then re-organized based on emerging themes during the analysis.

The first stage of the analysis was to identify all mention of aspects related to human health within each agenda text in order to develop an initial coding framework. This is a process called inductive analysis and involves developing descriptive labels (or ‘codes’) for each identified mention to health [27]. The next step involved comparing codes between all four agendas, to identify similar codes which could be grouped further into ‘sub-themes’ and overarching ‘themes’. This process was conducted using latent content analysis, where the codes were interpreted within the context of the agenda texts, seeking to understand the underlying meaning instead of using the exact phrasing [28]. All codes and themes derived through this process were included in the final analysis.

Given there were three different types of themes emerging, the coding framework was then organized into three broad themes: (i) themes related to health system strengthening—Using the World Health Organization’s building blocks of health systems framework [29], (ii) health outcomes, and (iii) themes related to health determinants, separate from health systems. Once the coding framework was re-organized, all four agendas were analysed again through a deductive content analysis process, where each agenda was analysed using the coding framework, to ensure all aspects of health that related to the framework had been captured. This process of decontextualizing and recontextualising data several times during analysis is an important aspect of qualitative research to maintain quality and trustworthiness in the process [28]. To further increase validity, two of the authors analysed the same data and compared codes and themes, a process known as triangulation [27,28]. All decisions were made by consensus. The final stage of the analysis involved analysing all four agreements for references that highlighted links between each other. The use of both deductive and inductive approaches can be helpful given that inductively built models can be complemented and developed further with the aid of deductive analysis [30].

## 3. Results

The three themes: (i) health systems (ii) health outcomes, and (iii) health determinants, and the corresponding sub-themes are presented in Table 1. The coding for the first sub-theme—Health systems—Was guided by the World Health Organisation (WHO)’s building blocks of health systems, which are the six fundamental building blocks identified as essential to strengthen health systems [29]. The second theme—Health outcomes—Consisted of multiple codes grouped into four broad sub-themes that reflected the content analysis (i) child and maternal health/mortality, (ii) sexual and reproductive health, (iii) infectious diseases, and (iv) non-communicable diseases (NCDs). The third theme—Health determinants—Consisted of three sub-themes; (i) sexual and reproductive rights, (ii) food security, and (iii) environmental health.

**Figure 1:**
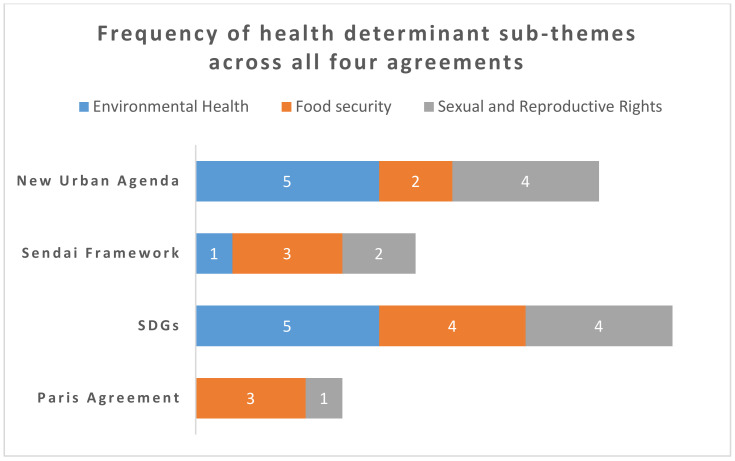
Overview of health determinant analysis across all four policy agreements.

For each of the three themes, a summary table (Table 2, Table 3 and Table 4) highlights which sub-themes were mentioned in each agenda. In addition, Figure 1 provides an overview of the health determinants theme analysis, to highlight the areas where the greatest synergies were found across the agendas. Only two sub-themes from the theme ‘health determinants’ were mentioned in all four agendas: sexual and reproductive rights, and food security. The codes that made up these two sub-themes are therefore presented (Table 5) and discussed further to identify which aspects were most aligned. Finally, the links between the four agreements are presented (Table 6) and discussed to identify opportunities for coherent implementation.

The results will be presented broadly across the four agendas, followed by a closer examination of results for each agenda, and concluding with a discussion of the health links between the four agendas. Table 1 summarises the themes and the corresponding sub-themes identified under the three themes.

### 3.1. Health Systems

The WHO’s health systems framework acknowledges the importance of strengthening health systems in order to achieve equitable, sustained improvements across health services and health outcomes. The six fundamental building blocks of health system strengthening requires partnership and collaboration across multiple sectors and is intended to improve overall health of countries, enabling progress towards global health targets [29]. At least one aspect of the six building blocks of health systems was mentioned by all agendas except the Paris agreement (Table 2). Only the SDGs referenced all six building blocks.

#### 3.1.1. Paris Agreement

The Paris Agreement only mentions health with reference to the obligation of all Parties when addressing climate change to respect, promote and consider the right to health as well as all other human rights. There is no specific mention of health systems strengthening, despite the recognition of the need for a collaborative and intersectoral approach. For instance, article 6 highlights the importance of integrated, holistic approaches to *“assist in the implementation of their nationally determined contributions, in the context of sustainable development and poverty eradication, in a coordinated and effective manner, including through, inter-alia mitigation, adaptation, finance, technology transfer and capacity-building as appropriate”*. However, no references to health or health systems particular were made.

#### 3.1.2. SDGs

The SDGs specifically advocate for a substantial increase in health financing (under SDG3), as well as recruitment, development, training and retention of health workforce. This agenda also highlights the importance of universal health coverage, including financial risk protection, access to quality essential health-care services, and access to safe, effective, quality and affordable essential medicines and vaccines for all. Subsequently, universal health coverage has emerged as a unifying umbrella within the SDG targets as many countries have made the political choice to pursue universal health coverage as an overarching goal for their health sector. However, many of these commitments are concentrated under SDG 3 on good health and wellbeing, with a few others that fall under other goals, such as SGD 5 (universal access to sexual and reproductive health).

#### 3.1.3. Sendai Framework

The Sendai Framework highlights the importance of multisectoral collaboration in building capacity within the health workforce in the field of disaster medicine, and supporting the development of community health workers to integrate disaster risk reduction approaches into health programmes. The World Health Organization’s International Health Regulations (2005) [31] is referenced multiple times within the Framework.

Health is framed as an essential component within the design and implementation of inclusive policies and social protection mechanisms. There is an emphasis on community engagement, and empowerment of those who are likely to be disproportionately affected by disasters. The Framework also highlights the importance of resilient infrastructure, including functioning hospitals and other health facilities, in order to provide life-saving and essential services.

#### 3.1.4. New Urban Agenda

Within the commitment to fostering healthy societies by promoting access to adequate, inclusive and quality public services, the Agenda includes healthcare facilities and services. Within the commitments of the Agenda it specifies a commitment to promoting the development of integrated housing policies and approaches across all sectors, including healthcare, in order to ensure access to affordable, adequate services. It also has a commitment to promoting equitable and affordable access to healthcare infrastructure and services.

### 3.2. Health Outcomes

Table 3 displays the analysis of the sub-themes under health outcomes (maternal, newborn and child health/mortality, sexual and reproductive health, infectious diseases and non-communicable diseases) across all four agreements.

#### 3.2.1. Paris Agreement

The Paris Agreement does not make any explicit references to health outcomes, but highlights the importance of promoting and considering the right to health.

#### 3.2.2. SDGs

The 2030 Agenda for the SDGs is the most comprehensively health focused of all four policy documents. SDG 3 *“Good Health and Well-Being”* is specifically focused on health. Targets within SDG3 encompassed all the health indicators coded in this analysis, and included ending epidemics such as AIDS, tuberculosis, malaria, neglected tropical diseases, combating hepatitis, water-borne diseases and other communicable diseases. Goal 3 includes commitments to universal access to sexual and reproductive health services, including family planning, information and education, and the integration of reproductive health into national strategies and programmes. It also focuses on prevention and treatment of non-communicable diseases including behavioural, neurological and development disorders, reducing death and disability from road traffic accidents, and substance abuse.

#### 3.2.3. Sendai Framework

The Sendai Framework discusses the negative impacts of disasters on health and the importance of disaster risk reduction to protect health. Maternal and child health, and sexual and reproductive health, are specifically mentioned when highlighting access to basic healthcare services. When enhancing disaster preparedness at the national and local level, the importance of recovery schemes to provide psychosocial support and mental health services is highlighted. Access to life-saving services for people with life-threatening and chronic diseases is mentioned under Priority 3: Investing in disaster risk reduction for resilience.

#### 3.2.4. New Urban Agenda

The vision of the New Urban Agenda includes ensuring healthy cities to foster prosperity and quality of life for all. Adopting healthy lifestyles, and access to healthcare services, are mentioned as aspect of ensuring environmental sustainability. In order to achieve this vision, the specified interlinked principles include ending the epidemic of AIDS, tuberculosis and malaria. Within healthcare services, specific reference is made to universal access to sexual and reproductive health services as an essential component to reduce newborn and maternal mortality. In addition, it commits to promoting equitable and affordable access to healthcare and family planning.

### 3.3. Health Determinants

Health determinants comprise the multiple factors that influence the health of individuals and communities [32]. In addition to factors that influence health systems, these broader determinants of health include socio-cultural, political and environmental factors that affect health and contribute to health inequities. The three sub-themes identified under health determinants were sexual and reproductive rights, food security and environmental health (Table 4).

Two determinants (sexual and reproductive rights and food security) were mentioned by all four agendas. As such, these two sub-themes will be analysed further. In order to highlight the synergies, Figure 1 displays how many times the codes within each of the three determinants were mentioned across the four policy agreements.

The Guttmacher-Lancet commission on sexual and reproductive health and rights highlighted that “achieving sexual and reproductive health rests on realising sexual and reproductive rights, many of which are often overlooked” [33]. Sexual and reproductive health and rights includes the right to the highest attainable standard of sexual and reproductive health, the right to make decisions on our own bodies and our sexualities, and to do so free from discrimination, violence or coercion. For the purposes of this analysis we have kept the two separate, with sexual and reproductive health coded under health outcomes, and sexual and reproductive rights coded under the health determinants code.

Some of the codes that contributed to these themes fit across a number of them, highlighting the interlinked nature of the determinants of health. For example, Water, Sanitation and Hygiene (WASH) was included under the theme environmental health as well as under sexual and reproductive rights, as it was discussed with reference to both access to healthy, safe drinking water to prevent illness and reduce mortality, as well as to ensuring that the particular hygiene needs of women and girls are met.

As illustrated in Figure 1, all four agreements mentioned some aspects of the health determinants food security and sexual and reproductive rights. It also shows that food security was the strongest sub-theme as an aggregate total between all four agreements. In order to explore this further, Table 5 displays the codes within these two determinants, indicating which agreements addressed each code.

Under food security, sustainable agriculture, and food and nutrition, were mentioned by all four policy documents. Within sexual and reproductive rights, the only code that all four agreements addressed was gender equality.

#### 3.3.1. Paris Agreement

The Paris Agreement highlights the importance of promoting and considering the right to health, as well as gender equality, the empowerment of women and intergenerational equity. It emphasises the importance of gender-responsive policies and approaches. It also recognises food security and ending hunger as a fundamental priority and highlights the importance of ensuring sustainable food production.

#### 3.3.2. SDGs

The 2030 Agenda affirms that ‘realizing gender equality and the empowerment of women and girls will make a crucial contribution to progress across all Goals and targets’. SDG 5 “Gender Equality” is specifically focused on gender-related issues, including elimination of all forms of violence against women and girls, eliminating harmful practices such as female genital mutilation (FGM) and child, early and forced marriages, and promoting women’s full and equal participation at all levels. It makes specific reference to SRHR as it pertains to the Program of Action of the International Conference on Population and Development (ICPD) and the Beijing Platform for Action.

The 2030 Agenda also has a focus on the specific and unique needs of women and adolescent girls throughout. Within food security, this included not just ensuring safe, affordable and nutritious food, and ending all forms of malnutrition, but also addressing the nutritional needs of adolescent girls, and pregnant and lactating women. Within the WASH sector this included not just access to safe drinking water, but also adequate and equitable sanitation and hygiene, paying special attention to the needs of women and girls.

Within environmental health, the Agenda emphasises the adverse impacts hazardous chemicals and air, water and soil pollution have on health. It promotes the provision of green spaces and sustainable urban planning, with a focus on reducing adverse environmental impacts of cities including by paying special attention to air quality. In addition, the Agenda commits to promoting resilience and holistic disaster risk management at all levels.

SDG 2 “Zero Hunger” has a focus on food security—To end hunger, achieve food security and improved nutrition and promote sustainable agriculture. This includes targets to reduce malnutrition, stunting and wasting in children under 5 years of age, ensuring sustainable food production systems, and implementing resilient agricultural systems with strengthened capacity for adaptation to climate change.

#### 3.3.3. Sendai Framework

The Sendai Framework highlights the importance of disaster risk reduction in order to protect the health of persons, communities and countries. Food security, food and nutrition, eradicating hunger, and access to essential food supplies are all discussed within the implementation of DRR policies and social safety-net mechanisms. In addition, it states that women should be empowered to lead and promote gender-equitable approaches during response, recovery, rehabilitation and reconstruction phases.

#### 3.3.4. New Urban Agenda

Within their descriptions of safe, healthy, inclusive environments the Agenda highlights eliminating harmful practices against women and girls and ensuring gender-responsive policies and urban planning. The Agenda recognises the need to address multiple forms of discrimination, including women and girls, and a commitment to gender equality is specifically mentioned.

A clean environment and good air quality are specified in their commitment to fostering healthy societies. Green and nature-based solutions are highlighted to reduce negative impacts to physical and mental health, and the public health costs of urban heat island effects and air pollution are noted. The New Urban Agenda recognises that urban centres make them and their inhabitants vulnerable to adverse climate events and other disasters, water and air pollution and vector-borne diseases. It includes a commitment to facilitating sustainable management of natural resources to reduce these impacts, and to promote disaster risk reduction and management. Further commitments include universal access to safe and affordable drinking water, nutritious and adequate food, and sustainable food production.

### 3.4. Links between the Agreements

The agreements were also analysed for references that highlight links between them, in relation to health, health policy and multi-sectoral coordination. As the New Urban Agenda was adopted one year after the other three, content was analysed for either specific references to the agreements, or references to the guiding principles underpinning the agreements (Table 6).

SDGs and New Urban Agenda both discuss the importance of strengthening capacity for early warning, risk reduction and management of national and global health risks and climate change related impacts. Both SDGs and the New Urban Agenda specifically mention the Sendai Framework, highlighting the need to align integrated disaster risk reduction approaches with the Framework.

All three agendas mention the Paris Agreement when discussing the importance of addressing greenhouse emissions and taking action on climate change. The Paris agreement does not mention any of the other three, but does make multiple references to sustainable development and the eradication of poverty. It also discusses integrated approaches with aims to *“enable opportunities for coordination across instruments and relevant institutional arrangements”.*

Even though the New Urban Agenda was developed after the other three agreements, the SDGs discuss the crucial role of sustainable urban development in improving quality of life, and effective, sustainable urban planning is incorporated within the Sendai Framework as well.

All four agendas specify commitments for enhancing cooperation and collaboration in order to achieve targets. Enhancing public and private sector partnerships, engaging civil society, and enabling opportunities for multi-stakeholder coordination and partnerships are highlighted by all. Importantly, all four agreements expressed commitments to promote meaningful participation of all stakeholders, using inclusive, transparent, gender-responsive approaches.

The Sendai Framework makes specific commitments that include collaboration with health. Under global and regional level commitments for Priority 3, the Framework commits “to enhance cooperation between health authorities and other relevant stakeholders to strengthen country capacity for disaster risk management for health, the implementation of the International Health Regulations (2005) [31] and the building of resilient health systems”.

## 4. Discussion

This policy review has evaluated the inclusion of human health within the mandates of these four major international agendas, and to what extent synergies and policy coherence exist across these agendas in order to support an integrated approach to implementation. The overarching implications of the results highlight four main messages: (i) the importance of increased awareness of health systems strengthening as a helpful framework to guide the integration of health issues across the agendas, (ii) only two themes had synergies across the agendas—Food security and gender equality, (iii) the lack of a governance mechanism to support the integration of these four agendas to enable national (and sub-national) governments to more feasibly implement their ambitions, and (iv) the vital component of health leadership in order for these agendas to fully reflect health concerns in a way that is integrative and systems-based. Finally, planetary health is reinforced here as a relevant and timely concept that can support the urgent shift to a healthy planet and people.

### 4.1. Lack of an Integrated Approach to Health

In order to support sustainable development and human health and well-being it is imperative that multi-sectoral and cohesive health responses are developed and implemented. Three of the four agreements include aspects of the WHO building blocks framework for health systems strengthening, such as universal health coverage, access to quality healthcare, and the importance of inclusive, resilient health infrastructure. However, across the agendas there is a lack of specific mention of health systems strengthening, or the ‘health in all policies’ approach. The absence of these core public health principles across the agendas makes it challenging for decision-makers to align policy direction and responses with these principals and weakens their intention to be used as guiding frameworks.

While the Paris Agreement does not mention the WHO building blocks of health, it has a strong emphasis on a rights-based approach to addressing climate change, and recognises the obligation of Parties to respect, promote and protect the right to health.

The SDGs have the most comprehensive health focus out of all the agreements. The SDGs incorporate the specific and siloed approach to health that has dominated since the MDGs, while also acknowledging the integrated nature of health today, with the determinants of health laying in all three economic, social, and environmental pillars of the SDGs. Together with robust targets and indicators, the SDGs represents a robust framework for monitoring and evaluation of health globally.

The Sendai Framework includes health in its goal and emphasises the importance of advancing health emergency and disaster risk management to achieve positive health outcomes [34]. Similarly, the New Urban Agenda highlights the important links between health and decisions related to sustainable urban development, and health has been identified as a “pulse” of the Agenda [35]. The WHO and UN-Habitat have been supporting ways to integrate health in urbanisation planning [36]. The Paris Agreement was particularly bereft of health mentions, which risks missed opportunities to harness the urgency created under the Paris Agreement to promote the health co-benefits of climate change mitigation and adaptation [37].

Furthermore, many of the agendas analysed lacked a common framing highlighting the health co-benefits of addressing the threats related to climate and other environmental changes. In all, there is a uni-directionality in how health is approached. Reducing health impacts is the end-goal while the health benefits that come alongside that reduction, such as improved health and wellbeing related to reduced air pollution or adopting a healthy lifestyle, is not a focus in these agendas [38]. We argue that the notion of attaining health is a necessary precursor for attaining other goals such as mitigating climate change, protecting biodiversity, and stemming the tide of the COVID-19 pandemic.

### 4.2. Food Security and Gender Equality—Two Issues That Were Common across the Agendas

The synergy between all four agreements on the importance of food security and gender equality presents crucial opportunities for collaboration and alignment to advance global health outcomes. Gender equality and the empowerment of women and girls is the cornerstone of building sustainable, resilient and prosperous societies, and is one of the most efficient ways to reduce health inequities, improve health outcomes, and ensure effective use of health resources [39]. Likewise, with food security looming as a continuing threat to health, and projected to worsen under climate change (e.g., [40]), it is imperative that this is a priority issue across these four agreements to enhance health policy coherence. The opportunities for alignment are further strengthened by the fact that food insecurity is a gendered issue, with women being more vulnerable to all aspects of food security [41].

### 4.3. Governance Mechanisms to Encourage Cohesion across the Agendas Must Be Developed

The four agendas studied in this paper take place in parallel processes operating in silos, often with little to no coordination between them [42]. This is not particularly surprising, given the challenges of the health sector working collaboratively, even within only one of these contexts, such as climate change [43]. The inception of these international agreements would have been a powerful moment to encourage a comprehensive and consistent understanding of health challenges and the messaging of these to signatory countries.

The lack of any process to support the coherence of these agreements at an international level likely impairs the potential of implementing these policies in a synergistic manner at the national and sub-national levels. This lack of interaction is perhaps a symptom of the disparate and independent processes that were followed to develop the agenda as there is no overarching governance mechanism that draws all four agendas together. Forward-looking components in the four agendas can be used to increase synergetic and cross-cutting ambition. For example, with regards to the Paris Agreement, the cyclical nature of the NDCs mean that future NDCs can take account of cross-cutting elements to improve synergies and policy coherence [44].

Furthermore, national governments need to ensure that all levels of society share the responsibility of implementing the four agendas coherently. They also need to mainstream goals across the four agendas into their overarching national development strategies to ensure greater coordination across governmental departments and agencies [45]. With no clear and comprehensive understanding of the health linkages and interdependencies arising from climate change, urbanisation, disaster risk reduction and sustainable development, it is difficult for policymakers to take an integrated approach to policymaking, particularly one that puts health at the centre. Without an integrative perspective, the alignment of these agendas to achieve what are fundamentally common goals—focused on a healthy planet and healthy people—is undermined.

### 4.4. Health Leadership—A Key Ingredient to Promote Trans-Sectoral Policies

The health sector is uniquely placed to leverage synergies and promote the development of strong trans-sectoral policies. The sector has experience via the Health in All Policies initiative [5], which facilitates inter-sectoral responses to health challenges. This is a process that is supported by the WHO and has the potential to be used during the process of national-level translation of these international agreements.

Effective leadership is fundamental to support and enable a more integrative approach to health decision-making. In some cases this will require capacity building to learn how to work beyond the traditional silos of sectors, and the ability to listen and understand different forms of knowledge and potentially competing priorities and interests. For instance, a minor inclusion in the Paris agreement emphasizes the need to work at a national level to promote greater engagement between health and climate actors, such as country climate negotiators. Further, with sustainable urbanisation, planning and public health professionals can ensure that health promotion, disease prevention and better health equity through good territorial planning is a central component of the communicable and non-communicable disease reduction and management responses.

Health synergies across the agreements has the potential to be unifying theme that brings diverse stakeholders together, as health is not only an indicator for monitoring progress but also a fundamental driver and pre-requisite of sustainable development.

### 4.5. Opportunity to Align the Implementation of the Agendas Using the Concept of Planetary Health

All agendas refer to the Paris Agreement and the SDG Agenda (despite the Paris Agreement itself not referring to the other agendas). This reflects the global attention on broad sustainability and climate change ambitions. In line with this attention and given that climate change is the greatest global health opportunity of our time, it is crucial that health is highlighted as a key concept within these ambitions, which—As shown in this study—Is represented the strongest in the SDG Agenda.

There are already several initiatives that monitor specific environmental changes and exposures relevant to health (Global Observatory on Pollution and Health, UNEP World Conservation Monitoring Centre, Lancet Countdown on Health and Climate Change for example). The interlinked nature of health systems, health outcomes and the sociocultural and environmental determinants of health highlight the need to think beyond the building blocks of health systems. In order to achieve substantial health and environmental gains, a planetary health approach is essential to maximise coherence between these four agreements. Some efforts are already underway to link data of environmental planetary boundaries to human health outcomes [46]; ‘Planetary Health Watch’ attempts to integrate data in ways that would allow improved attribution of environmental changes to human health outcomes. However, to date, little attention has been paid to sociocultural determinants in such initiatives.

In the last five years there has been renewed focus on planetary health, recognising the need for collective action at all levels of society to address threats to human health, sustainability of our civilisation, and the natural and human-made systems that support us [47]. This focus on an inclusive and integrative understanding of the health of people and the planet is even more urgent now given the COVID-19 pandemic. In response to the pandemic, the WHO has recently released their Manifesto for a healthy recovery from COVID-19 [48]. This manifesto includes six ‘prescriptions’ for a healthy, green recovery—(i) protect and preserve the source of human health: nature; (ii) invest in essential services, including water and sanitation, and clean energy in healthcare facilities; (iii) ensure a quick healthy energy transition; (iv) promote healthy, sustainable food systems; (v) build healthy, liveable cities; and (vi) stop using taxpayers money to fund pollution.

The planetary health concept integrates all of the components within the four agendas, and has a focus on health equity related to socioeconomic, regional and gender factors. In order to utilise opportunities to align all four agendas and accelerate progress, further work could be pursued with country-level policymakers and practitioners to determine the practicability of a planetary health approach to support implementation.

## 5. Conclusions

The adoption of the SDGs in September 2015 capped off a tripartite of international agendas that year in areas of substantial significance for population health which also included the Sendai Framework for Disaster Risk Reduction; and the Paris Agreement to respond to climate change. The New Urban Agenda followed shortly after in 2016. Each of these four agendas present, in theory, great hope for progressing global health outcomes and reducing the inequitable distribution of the current burdens of disease. However, in order for these agendas to strengthen our national responses to current and emerging health risks, we need to better understand and assess how health is framed in these agreements, and to what extent an integrative understanding of health is incorporated into these agendas. If health is not considered as an interlinked, multi-sectoral and transdisciplinary element, then opportunities to progress efforts to improve population health across the globe will be hampered. This paper discusses each agreement and its health relevance, and proposes ways to strengthen the incorporation of a systemic approach to health and health responses in order to increase our resilience to contemporary global challenges. This is of value to national level implementation and operationalization and to the global health community for future advocacy around health and the role it plays in future global agreements.

## Figures and Tables

**Table 1:** Themes and sub-themes from the analysis.

Themes	Sub-Themes
Health Systems	Financing
	Service Delivery
	Health Information Systems
	Access to Essential Medicines
	Leadership/Governance
	Workforce
Health outcomes	Child and Maternal Mortality/Health
	Sexual and Reproductive Health
	Infectious Diseases
	Non-communicable Diseases (NCDs)
Health determinants	Sexual and Reproductive Rights
	Food Security
	Environmental Health

**Table 2:** Analysis of WHO building blocks across all four agreements.

Theme and Associated Sub-Themes				
Health Systems	Paris Agreement	SDGs	Sendai DRR	New Urban Agenda
Financing	✕	✓	✕	✕
Service Delivery	✕	✓	✓	✓
Health Information Systems	✕	✓	✕	✕
Access to Essential Medicines	✕	✓	✕	✕
Leadership/Governance	✕	✓	✓	✓
Workforce	✕	✓	✓	✕

✓ indicates that the agreement mentions the theme, ✕ indicates it does not mention the theme.

**Table 3:** Analysis of health outcomes across all four agreements.

Health Outcomes	Paris Agreement	SDGs	Sendai DRR	New Urban Agenda
Maternal, newborn and child health/mortality	✕	✓	✓	✓
Sexual and reproductive health	✕	✓	✓	✓
Infectious diseases	✕	✓	✕	✓
Non-communicable diseases	✕	✓	✓	✓

**Table 4:** Analysis of health determinants across all four agreements.

Health Determinants	Paris Agreement	SDGs	Sendai DRR	New Urban Agenda
Environmental Health	✕	✓	✓	✓
Food Security	✓	✓	✓	✓
Sexual and Reproductive Rights	✓	✓	✓	✓

**Table 5:** Analysis of sexual and reproductive rights and food security sub-themes across all four policies.

Sub-Themes	Codes	Paris Agreement	SDGs	Sendai DRR	New Urban Agenda
Sexual and reproductive rights	Gender Equality	✓	✓	✓	✓
	WASH	✕	✓	✕	✓
	Harmful practices—Female Genital Mutilation/Child, Early and Forced Marriage	✕	✓	✕	✓
	Sexual Harassment, exploitation and Sexual and Gender-Based Violence (SGBV)	✕	✓	✓	✓
Food Security	Sustainable Agriculture	✓	✓	✓	✓
	Food and Nutrition	✓	✓	✓	✓
	Ending Hunger	✓	✓	✓	✕

**Table 6:** Referenced links between the agreements.

		References			
		SDGs	New Urban Agenda	Sendai DRR	Paris Agreement
**Agreements**	SDGs		✓	✓	✓
	New Urban Agenda	✓		✓	✓
	Sendai DRR	✓	✓		✓
	Paris Agreement	✓	✕	✕	
